# Exploring Virulence Factors and Alternative Therapies against *Staphylococcus aureus* Pneumonia

**DOI:** 10.3390/toxins12110721

**Published:** 2020-11-18

**Authors:** Jelle Vlaeminck, Dina Raafat, Kristin Surmann, Leen Timbermont, Nicole Normann, Bret Sellman, Willem J. B. van Wamel, Surbhi Malhotra-Kumar

**Affiliations:** 1 Laboratory of Medical Microbiology, Vaccine and Infectious Diseases Institute, University of Antwerp, 2610 Antwerp, Belgium; Jelle.Vlaeminck@uantwerpen.be (J.V.); leen.timbermont@uantwerpen.be (L.T.); 2 Department of Immunology, Institute of Immunology and Transfusion Medicine, University Medicine Greifswald, 17475 Greifswald, Germany; dina.raafat@med.uni-greifswald.de (D.R.); Nicole.Normann@med.uni-greifswald.de (N.N.); 3 Department of Microbiology and Immunology, Faculty of Pharmacy, Alexandria University, Alexandria 21521, Egypt; 4 Department of Functional Genomics, Interfaculty Institute for Genetics and Functional Genomics, University Medicine Greifswald, 17475 Greifswald, Germany; kristin.surmann@uni-greifswald.de; 5 Microbiome Discovery, Microbial Sciences, BioPharmaceuticals R & D, AstraZeneca, Gaithersburg, MD 20878, USA; bret.sellman@astrazeneca.com; 6 Department of Medical Microbiology and Infectious Diseases, Erasmus Medical Center Rotterdam, 3015 Rotterdam, The Netherlands; w.vanwamel@erasmusmc.nl

**Keywords:** *Staphylococcus aureus*, pneumonia, virulence, therapeutics

## Abstract

Pneumonia is an acute pulmonary infection associated with high mortality and an immense financial burden on healthcare systems. *Staphylococcus aureus* is an opportunistic pathogen capable of inducing *S. aureus* pneumonia (SAP), with some lineages also showing multidrug resistance. Given the high level of antibiotic resistance, much research has been focused on targeting *S. aureus* virulence factors, including toxins and biofilm-associated proteins, in an attempt to develop effective SAP therapeutics. Despite several promising leads, many hurdles still remain for *S. aureus* vaccine research. Here, we review the state-of-the-art SAP therapeutics, highlight their pitfalls, and discuss alternative approaches of potential significance and future perspectives.

## 1. Introduction

Pneumonia is one of the leading causes of death in developed countries, with up to 2.56 million recorded fatalities in 2017 [1]. Its immense impact on healthcare is even more pronounced among young children, where it accounts for 15% of all fatalities in children below five years of age [2]. Data from the United Nations Children’s Fund (UNICEF) estimates that every 39 s, a child succumbs to pneumonia [3]. Pneumonia is defined as an acute infection of the lung parenchyma caused by one or several co-infecting pathogens, which can be of fungal, viral, or bacterial origin [4,5]. In this review, we focus on bacterial pneumonia, where severe episodes are associated with high mortality rates, reaching up to 22% among ICU patients (all common pathogens) [6].

Based on the presumed origin of the pathogen, episodes of pneumonia are generally classified into four types that are sometimes difficult to differentiate: (i) hospital-associated pneumonia (HAP), constituting about 20% of all nosocomial infection [7], with an onset later than 48 h after hospital admission; (ii) ventilator-associated pneumonia (VAP), occurring after more than 48 h following intubation; (iii) community-associated pneumonia (CAP), with an onset before or within the first 48 h post-admission [8,9]; and (iv) health-care-associated pneumonia (HCAP), occurring in patients coming from high-risk environments such as nursing homes and extended-care facilities, in patients receiving long term care (e.g., dialysis), or patients who had undergone recent hospitalization prior to the current hospital admission [10]. CAP is the most common type of pneumonia and is commonly caused by *Streptococcus pneumoniae,* although other pathogens, both Gram-positive and Gram-negative, might also be involved [11] (Table 1).

## 2. Staphylococcus aureus Pneumonia

*Staphylococcus aureus* is a rare CAP-causing pathogen, as was shown by a longitudinal study involving nearly 10 million pneumonia cases requiring hospitalization, where in only 1.08% of the cases, *S. aureus* pneumonia (SAP) was identified as the primary diagnosis [17]. In HAP and VAP, *S. aureus* has a more prominent role as the pathology differs from CAP; the importance of *S. aureus* as a pneumonia pathogen was recently demonstrated in the ASPIRE-ICU observational trial [18]. However, despite a low prevalence in CAP, SAP, in general, is linked to a high case-fatality rate, ranging from 48% to 84% [19,20,21]. A recent retrospective analysis of 98 SAP patients admitted to a Spanish hospital between the years 2000 and 2014 revealed a 30-day mortality rate of 46.9% [22]. Also, SAP was recently identified as an important part of complications and mortality in SARS-CoV-2 patients [23]. This situation is even more complicated by the multidrug-resistant character of *S. aureus*. The longitudinal study of Jacobs et al. also showed that, of those above-mentioned patients hospitalized with SAP, approximately 78% of the cases were attributed to methicillin-resistant *S. aureus* (MRSA) [17]. MRSA clones harbor the staphylococcal cassette chromosome *mec* (SCC*mec*), a mobile genetic element conferring resistance to cefoxitin and many other β-lactam antibiotics [24]. Initially, pulmonary infections with MRSA were limited to HAP and VAP, and hence arose the term hospital-associated MRSA (HA-MRSA), such as the HA-MRSA sequence type (ST)239 lineage [25]. However, in the last two decades, there has been a surge in community-associated MRSA (CA-MRSA), which, in turn, is associated with CAP [26]. CA-MRSA infections became increasingly prevalent due to the global spread of highly virulent clones such as the USA300 (ST8) [27]. However, the distinction between HAP and CAP based on the causative clone has become increasingly difficult, since several CA-MRSA clones have been observed to cause nosocomial infections [9,28,29]. 

Apart from certain representative clones, HA- and CA-MRSA strains can be distinguished by specific molecular characteristics [28], for instance, by the type of SCC*mec* they carry. While HA-MRSA typically harbor the larger SCC*mec* types I, II, and III, CA-MRSA carry the smaller SCC*mec* types IV and V [28,30,31]. SCC*mec* also allows for the integration of other resistance-conferring genes into the three adjoining regions of the element [30,31]. Examples are (i) TetA(K) and TetA(L) efflux pumps, conveying resistance to tetracyclines whose genes have integrated in the SCC*mec* III cassette and (ii) integration of aminoglycosides resistance genes *aacA-aphD* in SCC*mec* II [32,33]. Such molecular events were largely responsible for the significant surge in multidrug-resistant *S. aureus* clones over the last decades. On the other hand, while CA-MRSA strains are susceptible to more antibiotic classes, they are more virulent due to the higher abundance of virulence factors, such as Panton-Valentine leukocidin (PVL), and a higher expression of core virulence factors [34,35]. Given the high levels of multidrug resistance, alternative anti-infective therapeutics have recently garnered much attention, in the hope of combating *S. aureus*. This review will therefore include a comprehensive overview of *S. aureus* virulence factors that have been targeted in search of an effective SAP vaccine, as well as outline some alternative therapeutic approaches that may hold promise for *S. aureus* research.

SAP occurs when the bacterium gains access to the epithelial cells of the upper airway due to damage of the mucosal lining, which can be facilitated by viral infections such as influenza and measles [36,37] or by the insertion of an endotracheal tube for mechanical ventilation. This exposure subsequently leads to the activation of the host’s proinflammatory innate immune response, with immune cell activation as well as neutrophil and macrophage recruitment (reviewed in [38]). The increased neutrophil influx into the lungs, accompanied by their secreted granular toxins, contributes to the lung pathology during SAP, by causing inflammation and necrosis of the lung epithelial cells [39,40,41]. Furthermore, the adaptive immune response further exacerbates the lung pathology. Indeed, a study in mice lacking CD4^+^ T cells showed improved bacterial clearance during SAP [39]. The complex interplay between *S. aureus* and the adaptive immune response is reviewed in [42], while the immunopathogenesis during pneumonia is reviewed in [43]. Apart from the role of the immune response, the contact between *S. aureus* and the upper airway epithelial cells is also believed to cause an upregulation of the bacterial virulence factors [44]. These virulence factors serve various functions, including cell lysis through lytic toxins, immune modulation as well as biofilm formation since several *S. aureus* clones have been identified as prolific biofilm formers, adding to the pathology [45]. Given their importance in SAP pathogenesis, which in turn is linked to a high case-mortality rate, those virulence factors, together with their molecular detection and potential as a therapeutic target, are the focus of this review. 

*S. aureus* is known to express a large number of virulence factors, ranging from adhesins to toxins to immune evasion proteins, and hence, targeting these factors has been the focus of modern anti-*S. aureus* therapy [46,47]. Several virulence factors of *S. aureus* have been studied in the context of pneumonia. The aim of researchers was to find a suitable antigen with the following characteristics: (i) is relevant to *S. aureus* pathogenesis in pneumonia; (ii) is conserved in many *S. aureus* strains; (iii) can elicit a protective immune response; and (iv) shows promising results both in vitro and in vivo in several pneumonia models.

## 3. Contribution of OMICs Techniques to SAP Research

Modern OMICs technologies have provided multiple tools to extensively research SAP and its relevant virulence factors.

### 3.1. Genomics

With the introduction of the whole genome sequencing (WGS) technology in the field of clinical microbiology, more information became available on *S. aureus* and its virulence factors. The first two *S. aureus* genomes were sequenced in 2001 by Kuroda et al., which provided completely new insights into the genomic background of this pathogen [48]. These and future studies revealed that the core genome is quite conserved across the *S. aureus* species, with variations mostly being attributed to mobile genetic elements such as transposons, insertion sequence elements, SCC*mec*, etc. Shortly after the Kuroda et al. study [48], the sequenced genome of *S. aureus* MW2 revealed that it harbored the *lukF* and *lukS* genes, encoding the highly potent PVL, on a prophage element integrated into the genome [49]. Over the next two decades, large-scale sequencing projects shed light on HA-MRSA and their high level of antibiotic resistance, the dynamics of CA-MRSA and the impact of horizontal gene transfer (reviewed in [35,50,51]). *S. aureus* genomics has been extensively reviewed [52,53].

### 3.2. Proteomics

Proteomics provided a valuable tool to understand the interactions of an infected host with the pathogen, helping to refine and further focus on virulence factors unexpressed in the early phases of the disease. A mass spectrometry experiment typically provides quantitative data of host and pathogen proteins and have been, until now, mostly performed in simple cell culture models using human lung cell lines to simulate single events during SAP [54,55,56]. Analysis of *S. aureus* proteins within a background of prevalent human proteins in in vivo samples remains, however, challenging. Nevertheless, in vivo studies already provided interesting results. For instance, in a murine *S. aureus* pneumonia model, host proteins found to be associated with infection included extracellular matrix proteins, as well as intracellular proteins such as phagosomal proteins and hemoglobin [57]. Another preliminary study, investigating *S. aureus*-influenza co-infection, reported distinct differences in host protein abundance levels, observed by two-dimensional difference gel electrophoresis (2D-DIGE), for about 200 proteins between co-infection or single-infection groups as well as non-infected control samples [58]. A global analysis of in vivo proteome adaptation of *S. aureus* in a mouse pneumonia model showed increased abundance of glycolysis-, amino acid biosynthesis- and fermentation-related proteins as well as virulence factors mediating oxidative stress response [56].

### 3.3. Metabolomics

Not only proteins but also metabolites are affected during infection, since the host and the pathogen need to share the intracellular resources. In a cell culture study, glucose and almost all amino acids were found inside the host cells at the time of infection [54]. In agreement with this observation, increased levels of proteins involved in bacterial glycolysis during the course of an infection reflect the consumption of this intracellular carbon source. Metabolomic analyses have been successfully employed to discover specific biomarkers that can distinguish CAP from other lung infections. Compared to patients suffering from chronic obstructive pulmonary disease (COPD), fewer phospholipids were found in the plasma of CAP patients [59]. More interestingly, metabolomics allows for the differentiation of pneumonia-causing pathogens. For instance, SAP is distinguishable from *S. pneumoniae*-induced pneumonia based on the host’s metabolite patterns [60]. Moreover, 25-fold lower concentrations of seven metabolites were observed in patients suffering from influenza-induced pneumonia compared to MRSA pneumonia [61]. In this review, we analyze potential targets, some of which are currently under development as potential therapeutics that have resulted from recent genomic, proteomic, and metabolomics studies (Table 2).

## 4. Targeting Virulence Factors Associated with Biofilms

An important factor that further adds to *S. aureus* pathology is the formation of biofilms [45]. A biofilm is defined as a microbially derived, sessile community embedded into an extracellular matrix, usually consisting of polysaccharides, proteins, nucleic acids and lipids [62,63,64]. It attaches to both biological (e.g., lung, intestine, heart valve, and tooth) as well as abiological surfaces, such as indwelling medical devices (e.g., catheters, implants). Biofilms have been implicated in various persistent human microbial infectious diseases, ranging from dental caries and periodontitis, to endocarditis, urinary tract infections, osteomyelitis, respiratory infections in cystic fibrosis patients, and ICU pneumonia [65]. This alternative phenotype is beneficial to bacteria since it allows them to evade multiple clearance mechanisms, such as antimicrobials and the host immune system, leading to treatment failure and recurrent/chronic infections [62,66]. *S. aureus* biofilm production and virulence are closely linked, since the main biofilm regulator, the accessory gene regulator Agr, is also vital for the expression of numerous virulence factors [67]. Several virulence factors are known to accumulate in in vivo *S. aureus* biofilms [68,69,70], and are thought to contribute to the structural integrity of the extracellular matrix [71]. Therefore, many of the biofilm-related virulence factors have been the target of research on *S. aureus* therapeutics (Table 2).

### 4.1. Haemolysins

#### 4.1.1. Alpha-Toxin (Hla)

Alpha toxin, also known as alpha-hemolysin (Hla), is a well-studied cytolytic protein. It is a member of the pore-forming beta-barrel toxin family and is encoded by the *hla* gene, which is one of the few virulence genes integrated into the core genome [109,110,111,112,113]. Hla lyses a plethora of cells including erythrocytes, epithelial cells, endothelial cells, T cells, monocytes, macrophages, and neutrophils. It can also stimulate a hyperinflammatory response, and disrupt epithelial and endothelial barriers, even at sublytic levels [110,114]. The importance of Hla as a virulence factor in SAP has been researched in mouse, rabbit and ferret pneumonia models [72,85,86,115], with studies reporting a disruptive effect on the air-blood barrier [116]. Moreover, isogenic *hla* mutants consistently demonstrated reduced infection severity compared to their wild type strains [85,114,117].

Apart from its role as a cytolytic protein, Hla also promotes biofilm formation, and hence plays an essential role during biofilm-associated infections, both at the attachment as well as the cell-cell binding stage [118,119]. Despite the specific pathways still being unclear, the importance of Hla in biofilm-related *S. aureus* pathogenesis was demonstrated in different models using human skin and porcine vaginal mucosal tissue [69,73], as well as using transcriptomic approaches [120]. The generation of high titers of natural antibodies (Abs) against Hla following invasive *S. aureus* infections, including pneumonia, further confirms these findings [103,121,122].

Because of its importance in *S. aureus* pathogenesis and SAP, Hla is often targeted as a treatment strategy. Hence, the protective potential of several anti-Hla Abs (e.g., ASN100, LC10) against pneumonia was tested in rabbit, murine, and ferret pneumonia models, where active as well as passive immunization showed promising results [72,74,75,76,77,78]. Recently, there have been several human trials evaluating the potential use of passive immunization with anti-Hla Abs in the protection against SAP. These trials involved anti-Hla monoclonal Abs (mAbs), such as suvratoxumab (AstraZeneca, Cambridge, United Kingdom) [79], AR-301 (Aridis Pharmaceuticals, San Jose, CA, United States) [80], and the multivalent antitoxin ASN100 (Arsanis, Inc., Waltham, MA, United States) that targets five different *S. aureus* pore-forming toxins (“7. Multicomponent vaccines”) [81]. Despite the fact that results from these trials did not reach statistical significance, passive immunization did show protective potential. For instance, AR-301 reduced the time spent on mechanical ventilation, whereas suvratoxumab reduced hospital and ICU duration as well as the duration of antibiotic treatment, while being safe and well-tolerated [79,80,81]. Apart from these mAbs, some natural compounds targeting Hla might provide a useful alternative for the prevention and/or treatment of SAP. These include the following: (i) a modified β-cyclodextrin compound, IB201, which was discovered based on its spatial similarity to Hla, and hence the hypothesis that it would block Hla activity with a high affinity [123]; (ii) bioflavonoid morin hydrate [124]; (iii) aloe-emodin, an active compound from aloe vera [125], and (iv) apigenin, an active compound from parsley [126]. All of these aforementioned compounds demonstrated adequate protection in murine SAP models [115,124,125,126].

#### 4.1.2. Beta- and Gamma-Toxin (Hlb & Hlg)

Other well-known toxins of *S. aureus* are beta-toxin (Hlb), a sphingomyelinase, and gamma-toxin (Hlg), a leukocidin present in nearly all *S. aureus* lineages. Leukocidins are a set of β-barrel pore-forming toxins, capable of targeting erythrocytes, endothelial cells and host immune cells, thus helping *S. aureus* to evade the immune system and induce an inflammatory response [127]. Both Hla and Hlb are prevalent among nearly all *S. aureus* lineages and have a distinct role in *S. aureus* biofilm formation and pathogenesis [69,128,129,130]. Research on the contribution of Hlb and Hlg to SAP cytopathology is scarce, and their overall role in *S. aureus* diseases is still poorly understood [131]. One study showed that mice infected with Hlb-deficient *S. aureus* exhibited less severe histopathological signs of pneumonia, including reduced levels of neutrophilic inflammation, vascular leakage and protein exudation [82]. Some multivalent, Hlg neutralizing Abs have been developed, and will be discussed later under “7. Multicomponent vaccines”.

### 4.2. Phenol-Soluble Modulins (PSMs)

Phenol-soluble modulins (PSMs) are comprised of seven amphipathic, α-helical peptides, namely PSMα1–α4, PSMβ1–β2, and the *S. aureus* delta-toxin, which are widespread among staphylococci [132,133,134]. PSMs are under direct control of the Agr quorum-sensing system, and they facilitate epithelial colonization and biofilm formation due to their surfactant properties [133]. Using a nonspecific, receptor-independent mechanism, and at micromolar concentrations, PSMs (especially PSMα) enable the escape of *S. aureus* not only from human neutrophils, but also from non-professional phagocytic cells, such as epithelial cells [133,135]. Although PSMs do not lyse cells at submicromolar concentrations, they can trigger an inflammatory host response in neutrophils by interacting with the formyl peptide receptor 2 (FPR2) thereby contributing to the destructive inflammatory response characteristic of bacterial pneumonia [83,133,134].

Several studies have reported a distinct role of PSMs in SAP. In a study using human lung epithelial cells, three PSMs demonstrated a dose-dependent cytotoxicity and interleukin (IL)-8 production [136]. Subsequent in vivo experiments using a murine *S. aureus*-influenza co-infection pneumonia model showed a significantly lower mortality rate in mice infected with a PSM-deficient mutant. Thus, PSMs contribute to a more severe outcome of pneumonia, highlighting their importance as a target for anti-infective therapy [136].

Hence, several approaches have been explored to target PSMs, in an attempt to counteract their pathologic function. Indirectly targeting PSMs by blocking the Agr system with an RNAIII-inhibiting peptide resulted in a decreased bacterial burden and a lower mortality rate in a mouse pneumonia model, along with reduced expression levels of *agr*, *psmα* and *psmβ* [83]. However, the protective effect of the RNAIII-inhibiting peptide was confined to neutrophils rather than macrophages. Indirect blocking of the transport system, the receptor binding or expression seems to hold more promise than neutralizing individual PSMs with mAbs because of the sequence diversity and functional redundancy of the seven PSMs [132].

### 4.3. Cell Wall-Anchored Proteins

Cell wall-anchored (CWA) proteins of *S. aureus* play a crucial role in its pathogenesis [137]. Several of those surface proteins have been associated with colonization of and adhesion to the respiratory epithelium in animal models of SAP [138,139]. 

#### 4.3.1. Fibronectin-Binding Protein A (FnBPA)

Fibronectin-binding protein A (FnBPA) is one of two highly conserved fibronectin-binding proteins that are expressed by *S. aureus* and is most frequently encountered in strains isolated from patients suffering from bacteremia or infective endocarditis [98]. Moreover, it can promote *S. aureus* biofilm formation via its A domain [140]. Due to its ability to attach to the respiratory epithelium, and its association with biofilm formation, FnBPA has also been associated with HAP and VAP [138,139]. 

In a recent study by Sharma-Kuinkel et al., patients with clinically cured SAP had higher IgM titers against FnBPA compared to patients with treatment failure, suggesting a protective role for anti-FnBPA Abs [103]. Based on this and other data, FnBPA emerged as an interesting virulence target in *S. aureus* research. Indeed, several Ab-based therapies against *S. aureus* biofilms have been attempted in preclinical studies, with FnBPA as a target molecule, and have been recently reviewed by Raafat et al. [141]. To date, there are no clinical trials involving FnBPA as a single protein vaccine [142]. However, FnBPA has been used in a novel bivalent fusion vaccine, SpA-D_KKAA_-FnBPA_37–507_ (SF), comprised of the D domain of staphylococcal protein A (SpA) and the A domain of FnBPA [98]. Active vaccination with SF in a murine pneumonia model reduced the bacterial burden in the lungs of infected animals, while passive immunization with rabbit polyclonal anti-SF IgG protected against *S. aureus* in a mouse bacteremia model.

#### 4.3.2. Staphylococcal Protein A (SpA)

SpA, a multifunctional CWA protein, promotes immune evasion by disturbing the opsonophagocytic clearance of a pathogen [143]. Furthermore, it is the only B cell superantigen produced by *S. aureus* [47]. The role of SpA in the pathogenesis of SAP has been attributed to its ability to bind tumor necrosis factor receptor 1 (TNFR1) on lung epithelial cells, leading to the activation of intracellular signaling, the expression of chemokines (such as IL-8), and the recruitment of neutrophils [144]. This increases inflammation of the airway epithelium, and thus, contributes to tissue damage [137].

SpA has long been regarded as a promising vaccine target for *S. aureus*. A non-toxigenic SpA variant, termed SpA_KKAA_, was developed, where each of the five Ig-binding domains of SpA was mutated to abolish its binding to the Fc portion of IgG and the V_H_3^+^ Fab fragments [145]. An anti- SpA_KKAA_ mAb, 3F6, was identified from mice immunized with SpA_KKAA_, and found to neutralize the Fcy and V_H_3^+^ Fab binding activity of SpA [146]. Passive immunization of neonatal mice with 3F6 protected them against *S. aureus* sepsis, increased their protective immunity against subsequent staphylococcal infections [99], and prevented nasopharyngeal colonization [100]. A human anti-SpA mAb, 514G3, derived from an individual with naturally occurring anti-SpA Abs, effectively opsonized *S. aureus* and successfully saved mice from *S. aureus*-mediated bacteremia [101]. 514G3 entered a phase I-II clinical study in patients with *S. aureus* bacteremia and exhibited a favorable safety profile [98,102]. Further research into 514G3 in other *S. aureus* infection models was implied, but no concrete results have been published so far [101].

#### 4.3.3. *S. aureus* Surface Protein X (SasX)

*S. aureus* surface protein X (SasX) is another CWA surface protein encoded predominantly in isolates found in Asia and Eastern Europe [84,137,147]. Its main function involves nasal colonization, but a role in biofilm formation and evasion of neutrophil killing has been reported [84,147,148,149,150]. The interest in SasX as a target was driven by the increased risk of colonized individuals to develop *S. aureus* infections [150], together with the higher incidence of *sasX* in HA-MRSA strains [148]. Vaccination with recombinant SasX led to an IgG1-dominated Ab response, as well as reduced acute lung injury and inflammation in a murine lung infection model. Interestingly, both active and passive immunizations reduced murine nasal *S. aureus* colonization [84].

#### 4.3.4. Staphylococcal Sortases (Srt)

Sortases (Srt) represent a family of membrane-associated bacterial enzymes that belong to a family of transpeptidases that is responsible for anchoring secreted proteins into the peptidoglycan layer of Gram-positive bacteria [151]. Within *S. aureus*, there are two Srts, SrtA and SrtB. SrtA, the more studied one, anchors several *S. aureus* polypeptides, including Spa, FnBPA and FnBPB, ClfA and ClfB [117,152] to the cell wall via their recognition (LPXTG) motif [153]. SrtB, on the other hand, anchors the iron-regulated surface determinant protein C (IsdC) to the *S. aureus* surface via its recognition sequence NPQTN [152,153].

In SAP, only SrtB has been studied so far although with contradictory results. Bubeck et al. reported that in a mouse lung infection model, an *S. aureus* Newman *srtB* deletion mutation only resulted in a small reduction in mortality [85]. On the other hand, in an in vitro study by Wang et al., the SrtB inhibitor baicalin, which by itself does not kill *S. aureus*, reduced the adhesion of *S. aureus* to human lung epithelial cells A549, thus reducing the *S. aureus*-induced damage to these cells, as well as reducing macrophage J774 inflammatory activity [154]. Blocking the attachment of a pathogen to the host tissue may provide a potential alternative adjunctive therapy to conventional antibiotics in infection control [155]. Since many substrates of SrtA and SrtB are known virulence factors involved in the cell adhesion of *S. aureus*, pharmaceutical inhibitors of Srts are considered promising candidates in anti-infective therapy [154,156,157]. However, the use of neutralizing mAbs against Srts has not been investigated so far. 

## 5. Other Explored *S. aureus* Pneumonia-Related Targets

### 5.1. Panton-Valentine Leukocidin (PVL)

Panton-Valentine leukocidin (PVL) is one of the best-described bi-component *S. aureus* toxins, since strains harboring the *lukSF-PV* gene have been linked to more severe and lethal infections [131]. Unlike the other leukocidin-encoding genes, *lukSF-PV* is not highly prevalent in the *S. aureus* population (5% prevalence) [158], but is largely associated with CAP-causing MRSA strains [159]. Upon infection, it causes the release of a so-called “cytokine storm” (an over-activation of the immune system leading to high inflammatory cytokine titers) in lung epithelial cells, leading to increased lung inflammation and damage due to necrosis [41,160]. In contrast to other leukocidins, PVL does not possess hemolytic activity, due to an alternative tertiary conformation, which is unable to recognize the Duffy antigen receptor [161]. Nevertheless, isolates harboring the PVL-encoding genes, *lukS-PV* and *lukF-PV*, have been linked to severe and fatal cases of necrotizing pneumonia (NP), making it an important target for drugs developed against SAP [162,163,164,165,166]. The role of PVL in the pathogenesis of NP and staphylococcal disease has been extensively reviewed in [167,168].

NP caused by PVL-harboring *S. aureus* can be treated with antibiotics (single or combination treatment), as proven by in vivo studies and several clinical case reports [169,170,171]. However, because of the high rates of antimicrobial resistance observed in various *S. aureus* lineages, other treatment options have been explored. For instance, treatment of polymorphonuclear leukocytes (PMNs) with commercial intravenous immunoglobulin (IVIG), often used in the treatment of severe *S. aureus* infections, was able to neutralize the cytotoxic effects of PVL. Further in vivo experiments using a rabbit SAP model showed a protective effect, both with IVIG alone and in combination with either vancomycin or linezolid [87]. IVIG treatment resulted in up to 100% reduction in mortality when treating CA-MRSA-induced pneumonia, whereas only 50% mortality reduction was observed when a HA-MRSA (USA100/NRS382) strain was used [87]. Attenuated PVL components (LukS-PV_T28F/K97A/S209A_ and LukF-PV_K102A_) provided protection in a mouse sepsis model, and lead to partial protection in a NP rabbit model [86,88]. A combination therapy based on these components is described under “7. Multicomponent vaccines”. Other mAbs targeting LukS-PV were developed for diagnostic purposes but were not tested for neutralizing capabilities [89]. Finally, since PVL induces a cytokine storm during pneumonia, anti-inflammatory drugs have also been under investigation to reduce its pathologic effects. Treatment with Anakinra (Swedish Orphan Biovitrum AB, Stockholm, Sweden), human IL-1 receptor antagonist protein used in rheumatoid arthritis [172], resulted in decreased cytokine production in a PVL-induced NP rabbit model. Unfortunately, Anakinra therapy did not show a comparable effect when probed in SAP [173]. 

### 5.2. S. aureus Extracellular Vesicles (SEVs)

*S. aureus* extracellular vesicles (SEVs) are lipid bi-layered nanoparticles (20–200 nm in size) containing cytosolic proteins, membrane proteins, peptidoglycan, lipoteichoic acid and many pathogenic molecules, such as enterotoxin (SEQ), IgG-binding protein (Sbi) and hemolysins [90,91,174]. Although the mechanism remains unclear, SEV-associated proteins can influence bacteria–host interactions during systemic infections, and induce a host immune response, albeit in a different way than secreted proteins. Vaccination with SEVs induced an adaptive immune response, as judged by serum SEV-reactive IgG and IgM levels and T cell responses [90,91,92,93]. Moreover, administration of SEVs increased the resistance of *S. aureus* to killing by whole blood or purified human neutrophils ex vivo and increased in vivo survival [90]. The effects of SEV immunization on SAP and mortality were studied, where immunized mice were protected against *S. aureus*-induced infection [92]. 

The structure of SEVs could provide a powerful and innovative strategy as drug delivery vehicle as well as vaccination agent with many advantages, including their nano-scale size, favorable toxicity profile, intracellular location, activation of both innate and adaptive immune systems and non-requirement of adjuvants [91,92,93].

### 5.3. Lipoteichoic Acid (LTA)

Lipoteichoic acid (LTA) is a major CWA of most Gram-positive bacteria. Structurally, it is a polymer of teichoic acids (TAs), which in turn consist of polyanionic repeating units bound together with phosphodiester bonds, that is linked to the outer leaflet of the cytoplasmic membrane via a glycolipid anchor [175]. LTA is an example of pathogen-associated molecular patterns [PAMPs], which are highly conserved motifs recognized by pattern-recognition receptors displayed by phagocytic cells involved in host defense, the Toll-like receptors (TLRs). Their recognition leads to the activation of intracellular signaling cascades, ultimately resulting in a proinflammatory response and activation of the innate immune system [176,177].

Recognition of PAMPs by TLRs is considered to be important for an appropriate immune response against pathogens that enter the lower airways. In vitro, LTA has potent proinflammatory and pro-apoptotic effects on human alveolar macrophages, which are the major effector cells in host defense against respiratory tract infections [94]. Moreover, LTA, acting via TLR2, elicits neutrophil recruitment into the pulmonary compartment both in mice as well as humans, ultimately resulting in pulmonary inflammation [95,96]. It also activates bronchoalveolar coagulation with concurrent inhibition of anticoagulant and fibrinolytic mechanisms, and thus affects hemostasis in humans [97].

Despite the fact that LTA is a crucial component of *S. aureus*, by itself, it is deemed unsuitable as a vaccine candidate, since it is a thymus-independent antigen, which has weak immunogenicity, and which cannot sufficiently induce immune memory. This limitation might be overcome by alternatives, such as the conjugation of LTA as a polysaccharide antigen to a carrier protein, an approach that has been successfully implemented in a number of carbohydrate conjugate vaccines, such as the pneumococcal 13-valent (Prevenar 13^®^, Pfizer, New York City, NY, United States) and the *Haemophilus influenza* typa b (HiB) conjugate vaccines [178]. Another alternative was reported by Yi et al., where they identified a tetra-branched multiple antigenic peptide, named MAP2-3, that can mimic the epitope of LTA [179]. Immunization with MAP2-3 as a surrogate of LTA elicited humoral immune response (including high levels of functional LTA-specific IgG Abs), and protected mice from *S. aureus* systemic infection. Moreover, passive immunization with polyclonal anti-MAP2-3 sera mitigated acute lung injury in mice with pneumonia, and also proved useful in bacteremia and skin infection models [179].

## 6. Potential Unexplored Targets in *S. aureus* Pneumonia

### 6.1. Leukocidin AB (LukAB)

Leukocidin AB (LukAB; sometimes referred to as LukGH) was recently discovered, when isogenic mutants of *S. aureus* Newman, with disrupted AT-, Hlg- or leukocidin ED (LukED)-encoding genes, showed high cytotoxicity against human immune cells. Exoproteome analysis revealed LukAB as the main contributor to human phagocyte killing [180]. Besides the active killing of innate immune cells, LukAB also disrupts the adaptive immune response by directly killing dendritic cells, and by blocking dendritic cell-mediated activation and proliferation of CD4^+^ T cells [181]. Remarkably, it was found that LukAB not only enhances the formation of neutrophil extracellular traps (NETs), that immobilize pathogens and attract phagocytes, but also leads to nonspecific host tissue damage [182]. 

Despite the cytopathogenesis of LukAB, and the fact that several neutralizing mAbs have been developed, it has not yet been investigated in SAP models. Three LukAB-specific human mAbs, which were generated from human B cells, neutralized LukAB cytotoxicity, and improved bacterial clearance in a murine *S. aureus* sepsis model [105]. Moreover, neutralizing Abs were recently generated from two *S. aureus* strains with mutated *lukAB* genes (LukA_D39A_ and LukB_R23E_), which were able to block the CD11b cellular target, and prevent LukAB-dependent cell lysis in a human monocytic cell line [106]. These components were also tested as part of a multivalent toxoid vaccine, described under “7. Multicomponent vaccines”. Similar to what has been observed for PVL, commercial IVIG has been shown to contain neutralizing anti-LukAB [107].

### 6.2. Leukocidin ED (LukED)

Leukocidin ED (LukED) is another bi-component toxin, where the tertiary protein configuration of the core domains shows high structural similarity to those of other leukocidins [183]. The genes encoding the toxin subunits, *lukED*, are highly conserved in MRSA clones isolated across the world, with up to 85% carriage rates [131], especially among epidemic strains [184]. In addition to innate immune cells, LukED also kills cells that carry the CCR5 receptor found on myeloid and effector memory T lymphocytes; hence, CCR5-deficient mice are protected from LukED-mediated killing [108,185]. Therefore, analogous to LukAB, LukED is capable of disrupting the host’s innate as well as adaptive immune systems.

Given that LukED uses CCR5 as receptor, a potentially interesting treatment option was found in maraviroc (Pfizer, New York City, NY, United States), a small-molecule entry inhibitor of CCR5, used in the treatment of human immunodeficiency virus (HIV) infection. HIV and LukED share the CCR5 receptor as a cellular target, but bind to different sequence determinants [108,186]. Nevertheless, maraviroc was shown to render murine macrophages partially resistant to LukED-mediated killing [186], as well as to inhibit LukED functionality in human HEK293T and SupT1 cell lines [108]. 

### 6.3. Ferric Uptake Regulator (Fur)

Members of the ferric uptake regulator (Fur) family of transcription regulators are found in most Gram-positive and Gram-negative bacteria, and are involved in the regulation of iron and zinc metabolism [187]. Fur-mediated regulation of iron metabolism has been described in detail in a series of recent reviews [187,188]. While investigating the role of Fur in a murine SAP model, Torres et al. demonstrated that *S. aureus* lacking Fur was more susceptible to neutrophil-mediated killing. Furthermore, the bacterial load in animals infected with the wild type *S. aureus* was approximately 1.5-log greater than in those animals infected with the *fur* deletion mutant [104]. To our knowledge, Fur has not been investigated as a target for active or passive immunization, but since it is involved in the expression of many pneumonia-associated *S. aureus* virulence factors, such as Hla, HlgA, LukED, and LukSF, it might be a promising candidate for future vaccine studies.

## 7. Multicomponent Vaccines

The focus in the last couple of years has been increasingly shifting towards targeting multiple *S. aureus* virulence factors simultaneously. An increasing number of studies are providing evidence that vaccines consisting of two or more components have a higher protective potential in mouse models than those involving either of the individual proteins [98,189]. For instance, active immunization with the SF fusion vaccine (see “Section 4.3.1. Fibronectin-binding protein A (FnBPA)”) induced a strong Ab response, together with a Th1/Th17-polarized cellular response, and was thus more protective against different *S. aureus* strains in three different mouse models of infection (sepsis, skin infection and SAP), compared to either of the single components, highlighting the greater potential of multicomponent vaccine approaches [98]. Therefore, despite a seemingly long list of potential vaccine targets currently under consideration for both preclinical and clinical trials, most researchers support the notion that multicomponent vaccines might be the future of vaccine research [190,191,192].

**Figure 1:**
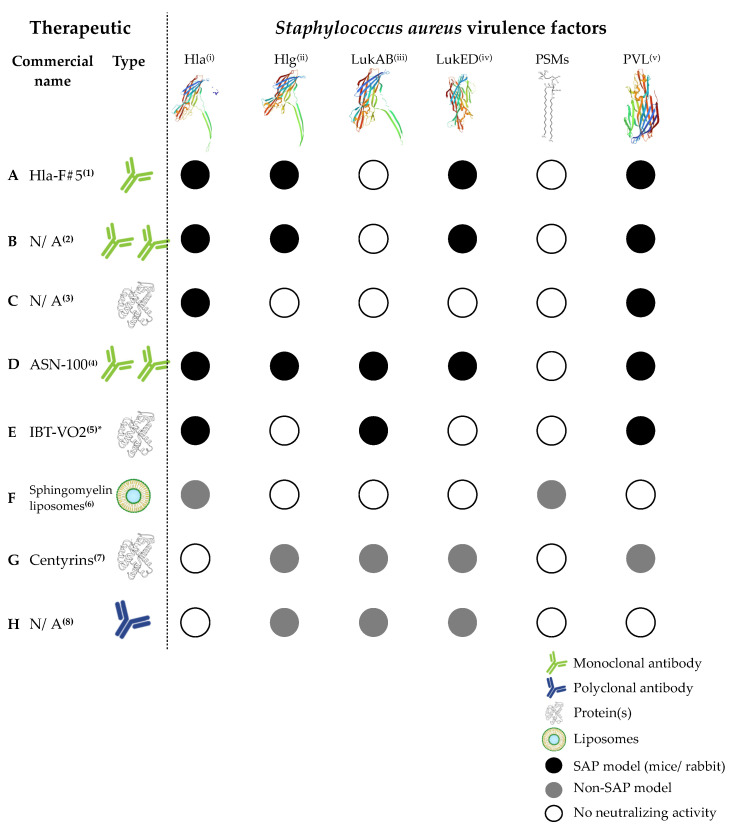
Therapeutics targeting multiple *S. aureus* virulence factors. The most commonly investigated virulence factors of *S. aureus* are Hla: Alpha-toxin; Hlg: Gamma-toxin; LukAB: Leukocidin AB; LukED: Leukocidin ED; PSMs: Phenol-soluble modulins and PVL: Panton Valentine leukocidin. Hla and PVL are often included in multivalent *S. aureus* vaccines, while PSMs are not. SAP: *S. aureus* pneumonia; N/A: not available. *: also neutralizes enterotoxins A and B and toxic shock syndrome toxin 1. (1): [193,194]; (2): [195]; (3): [86]; (4): [74,196]; (5): [197,198]; (6): [199]; (7): [200]; (8): [201]. (i): [202]; (ii): [203]; (iii): [204]; (iv): [183]; (v): [205].

An overview of the current *S. aureus* multicomponent vaccines, and their corresponding targets, is given in Figure 1. We will highlight some in the following paragraphs.

### 7.1. Therapeutics Neutralizing Multiple Leukocidins

A promising mAb tested in the context of pneumonia was Hla-F#5 (Figure 1A), which cross-reacts with Hla, gamma-hemolysin (HlgAB and HlgCB), LukED and PVL [193]. A study showed that passive immunization of mice suffering from SAP with this monoclonal Ab drastically increased their survival rate. Similar results were later obtained by Diep et al. in a rabbit pneumonia model [194]. 

More promising results by the Diep group came from two studies conducted in a NP rabbit model. The first study showed that passive immunization using a combination of an Hla-specific mAb (suvratoxumab) with a cross-neutralizing leukocidin mAb (SAN177 or SAN481) reduced mortality to 7% and 0%, respectively (Figure 1B) [195]. The second study showed similar results when attenuated Hla (Hla_H35L/H48L_), combined with two attenuated PVL components (described in [88]), were used for active immunization [86], where 100% protection from lethality was observed in immunized rabbits (Figure 1C). Besides Hla-F#5, the Nagy research group also developed a second combination of two mAbs, termed ASN100 (Figure 1D). ASN100 consists of ASN-1, which neutralizes Hla, PVL, LukED, and Hlg, as well as ASN-2, which is specific to LukAB. Administration of this Ab-combination protected human PMNs and white blood cells against lysis [196]. ASN100 was then further tested in rabbit pneumonia models, where passive immunization increased survival and protection against MRSA- and MSSA-induced pneumonia [74]. This led to a phase II interventional trial, where heavily *S. aureus*–colonized, mechanically ventilated patients were treated with ASN-100 (Clinical Trials Identifier: NCT02940626). However, there was no significant reduction in the incidence of SAP on day 22 (Table 3).

A final, interesting, multivalent vaccine currently under development is IBT-VO2 (Figure 1E). This heptavalent vaccine contains rationally designed Hla, PVL LukS, LukF, LukAB, enterotoxins A and B and toxic shock syndrome toxin 1 toxoids [197]. Due to structural similarities, the multi-subunit vaccine elicits an antibody response that is cross-reactive with 12–15 *S. aureus* toxins and protects from *S. aureus* disease in multiple mouse and rabbit infection models [198]. Following the current pre-clinical phase, IBT-VO2 will enter a phase I clinical trial and hence recently received extra financial support to advance its development [206].

### 7.2. Multitarget Therapeutics Involving PSMs

In an interesting approach, Wolfmeier et al. used sphingomyelin liposomes (Figure 1F), either with or without cholesterol, to neutralize secreted PSMs, together with other virulence factors, both in vitro (human *S. aureus* blood and epithelial cell infections) and in a murine dermonecrosis model [199]. Sphingomyelin liposomes prevented cell lysis by PSMs, especially PSMα3, in all cell types, whereas sphingomyelin liposomes containing cholesterol specifically sequestered Hla. 

Both liposome types reduced the extent of murine dermonecrosis [199]. It is noteworthy to mention that a mixture of both liposome types has recently been tested in a phase I clinical study against severe pneumococcal pneumonia (Clinical Trials Identifier: NCT02583373), but its usefulness in SAP is yet to be determined. Furthermore, it seems advisable to target PSMs and Hla simultaneously, since it was shown that PSMs regulate the production of Hla both in vitro and in vivo [207]. A mutant strain deficient for PSMα, PSMβ, and delta-toxin produced relatively lower amounts of Hla and exhibited reduced virulence in murine skin infection and pneumonia models [191].

## 8. Current Issues Hampering *S. aureus* Vaccine Research

Despite much research focused on targeting *S. aureus* virulence factors, no suitable vaccine is yet available. mAbs neutralizing single factors have been developed successfully, but many of the conducted in vitro studies did not reach clinical potential (Table 3). This is due to the redundancy of most of the above-mentioned virulence factors [201], as well as their complex regulatory mechanisms [208]. Targeting the regulatory factors like Fur and Agr might indeed be taken into consideration, as has already been shown in the case of PSMs [83]. 

However, one needs to keep in mind that Abs solely targeting *S. aureus* virulence factors might have a lower efficacy against HA-MRSA, despite the use of multicomponent vaccines. As mentioned, above HA-MRSA lineages harbor less chromosomal virulence factors and express them in lower quantities than CA-MRSA. Therefore, inhibiting those factors in HA-MRSA might prove to be ineffective. Also, there have been indications that an antibody-based therapy is more efficient in SAP patients with low *S. aureus* colonization levels [209], further complicating the use of Abs to treat SAP patients. However, prophylactic use of Abs could still be considered in said cases.

Another obstacle facing *S. aureus* vaccine research lies in the fact that many of the virulence-targeting therapeutics have been initially tested in murine SAP models. This is however a huge limitation, since some toxic virulence factors, such as Hla, leukocidins, and PVL, exhibit a high level of host tropism. Whereas PVL actively kills human and rabbit PMNs, murine PMNs are immune to its cytotoxicity [194]. On the other hand, LukAB shows a reduced activity towards rabbit and murine PMNs [210], which exhibit a lower affinity variant of the CD11b target [211]. The absence of appropriate small animal models could be overcome by the use of genetically modified (i.e., “humanized”) mice. Indeed, mice with a human hematopoietic system showed similar vulnerability to PVL as observed in studies performed in rabbits or human cell lines [212]. Alternatively, mouse-adapted *S. aureus* strains can be used to optimize murine in vivo models [213,214]. However, this might still prove to be difficult to transfer to human-infecting strains. Instead of adapting the model, a modification of the toxin might achieve a similar goal. For instance, a recombinant LukAB protein showed equal cytotoxicity towards rabbit as well as human PMNs [215].

## 9. Alternative Therapeutic Strategies

### 9.1. Bacteriophages

The current worldwide epidemic of increasing antimicrobial resistance is accompanied by a greater understanding of the damage broad spectrum antibiotics have on the healthy microbiome. Hence, alternative approaches—other than antimicrobials—have lately garnered the attention of researchers, in an attempt to treat multidrug resistant pathogens. One such alternative, which has also been explored in the context of SAP, is phage therapy, where bacteriophages are used to selectively target the infecting pathogen. In one study involving mice with lung-derived septicemia, treatment with *S. aureus* φS13′ increased murine survival from 10% to 67% [216]. Another randomized, blinded, controlled in vivo experimental study compared the efficacy of phage therapy versus teicoplanin treatment in rats with MRSA-induced VAP [217]. The administration of a four phage cocktail, increased rat survival to 58%, comparable to teicoplanin treatment (50%). In another study, conducted by the same research group and published in 2020, rats with MRSA-induced VAP were treated prophylactically with nebulized bacteriophages, resulting in increased survival (70% versus 0% in the control group) as well as a reduced bacterial burden [218].

Another promising treatment option involves the bacteriophage cocktail AB-SA01 that contains three myoviruses related to *Staphylococcus* φK [219]. The product killed 94.5% of the 205 clinical *S. aureus* isolates tested in vitro, which included both MRSA as well as vancomycin-intermediate *S. aureus* (VISA) [219]. Moreover, in a BALB/c mouse acute pneumonia model, it was shown that bacterial clearance in the lungs of AB-SA01-treated mice was comparable to that in mice treated with vancomycin. An additional benefit of AB-SA01 over antibiotics is that the frequency of spontaneous resistance was found to be low. In clinical testing, AB-SA01 was deemed safe and well-tolerated when administered intranasally in patients suffering from chronic rhinosinusitis, and thus became the first clinical trial with intravenous phage therapy to be approved in the USA [220]. In an ensuing single-arm, non-comparative clinical trial, intravenous administration of AB-SA01 in 13 patients with severe *S. aureus* infections, including endocarditis and septic shock, did not show any adverse reactions. More interestingly, eight patients survived and showed clinical improvement after 14 days of treatment [221]. Further controlled clinical trials are to be expected.

Besides using the phage itself for treatment, other studies explored a possible use of phage-derived lysines. A 2015 study investigating the efficacy of LysGH15 showed that treatment with the lysine achieved 80% survival in mice with SAP. The survival rate further increased to 100% when LysGH15 was combined with apigenin, a naturally occurring flavonoid [222]. In a second study, conducted in 2018, phage-derived endolysin SAL200 was tested in murine SAP, and achieved higher survival, lower bacterial burden, and improved lung histopathology compared to the control group [223].

Benefits of phage therapy are the little to no adverse host reactions, generally low frequency of spontaneous resistance against the bacteriophage, and their ability to target and kill multidrug resistant clones [219]. However, phage therapy also has its limitations, including strain specificity and the potential for immunogenicity preventing repeat dosing [224].

### 9.2. Outer Membrane Vesicles (OMVs)

Another interesting approach exploits bacterial outer membrane vesicles (OMVs) as a vehicle for delivering foreign antigens into the host, with the aim of eliciting protective immune responses. For instance, Irene et al. expressed five highly conserved *S. aureus* vaccine candidates (Hla_H35L_, SpA_KKAA_, FhuD2, Csa1A, and LukE) in *Escherichia coli* as fusions to a lipoprotein leader sequence. The lipoproteins were compartmentalized in OMVs and elicited high antigen-specific Ab titers in immunized mice. Moreover, the penta-valent OMV-based *S. aureus* vaccine protected mice from challenge with the *S. aureus* Newman strain in a sepsis model [225]. This suggests that those engineered OMVs could represent a valid alternative for the development of an efficacious *S. aureus* vaccine.

## 10. Concluding Remarks

In conclusion, much effort has been invested in exploring *S. aureus* virulence factors, thus improving our understanding of their mode of action and their role in SAP pathogenesis (Table 2). Classical immunization strategies involving the use of Abs have led to the development of several promising therapeutics. However, many hurdles still remain in the path of an effective SAP vaccine given the intricate genetic background of *S. aureus* (Table 4). Finally, apart from classical methods of active and passive immunization, alternative approaches should be considered (Table 4). SEVs and OMVs could provide innovative ways of drug delivery, while phage therapy provides an interesting lead showing some success in different clinical settings. A systems biology approach combining data generated from genomic, proteomic, and metabolomic studies is necessary to further our knowledge on bacteria–host interactions. Moreover, much attention has recently been dedicated to the respiratory microbiome and its effect on pneumonia pathogenesis. These studies debate Koch’s postulates and raise the question that if one pathogen is suppressed, another might prevail. More research will hence be required in these directions in order to develop a safe and effective SAP therapeutic.

## Figures and Tables

**Table 1:** Pathogens commonly encountered in the different types of bacterial pneumonia.

Class	CAP ^(a)^ [11,12,13]	HAP/VAP [11,14]	HCAP [12,15]
Gram-positive	*Streptococcus pneumoniae* *Staphylococcus aureus* ^(b)^	*Streptococcus pneumoniae* *Streptococcus* spp. *Staphylococcus aureus* ^(b)^	*Streptococcus pneumoniae* *Staphylococcus aureus* ^(b)^
Gram-negative	*Mycoplasma pneumoniae Chlamydophila pneumoniae* *Haemophilus influenzae Legionella* spp. *Pseudomonas aeruginosa* Enterobacteriaceae (*Escherichia coli, Klebsiella* spp., *Enterobacter* spp., *Proteus mirabilis*)	Enterobacteriaceae (*Escherichia coli, Klebsiella* spp., *Enterobacter* spp., *Proteus* spp.) *Serratia marcescens* *Pseudomonas aeruginosa* *Acinetobacter* spp. *Haemophilus influenza* *Stenotrophomonas maltophilia*	*Haemophilus influenzae* *Pseudomonas aeruginosa* *Acinetobacter baumannii* *Stenotrophomonas maltophilia*

spp.: species; ^(a)^ pathogen identification fails in about 50% of cases [16]; ^(b)^ both methicillin-resistant (MRSA) and methicillin-sensitive *S. aureus* (MSSA).

**Table 2:** Summary of virulence factors explored as potential targets for *S. aureus* pneumonia therapeutics.

Targeted in SAP	Virulence Factor	Research Stage ^a^	Approach/Strategy	Main Results	Advantages of Targeting ^b^	Disadvantages of Targeting ^b^	References
Yes	Hla	PC	in vitro: tissue cultures in vivo: murine, rabbit, and ferret models	Protective potential of passive and active immunization	Core virulence factor; crucial role in SAP	Complex regulation	[69,72,73,74,75,76,77,78,79,80,81]
		C	Human clinical trials	Protective potential of passive immunization			
	Hlb/Hlg	PC	in vivo: murine SAP model	Hlb-deficient *S. aureus* shows less severe histopathology	Prevalent in nearly all *S. aureus* lineages; distinct role in pathogenesis	NA	[82]
	PSMs	PC	in vivo: murine SAP model	Reduced mouse mortality by indirect targeting (blocking Agr system)	Distinct role in SAP	Neutrophils are more protected than macrophages	[83]
	SasX	PC	in vitro: cell culture; vaccination studies in mice (skin abscess and lung infection model)	Induction of antigen-specific IgG response; protection from *S. aureus*-induced infection and colonization	(i) active immunization induced IgG1 response and reduced acute lung injury; (ii) active and passive immunization reduced *S. aureus* colonization; (iii) anti-SasX IgG increased *S. aureus* killing by human neutrophils	NK	[84]
Yes	Srt	PC	in vitro: cell culture in vivo: murine *S. aureus* lung infection models	Deletion of SrtB reduced mortality; reduced *S.aureus* adhesion to human lung epithelial cells	Anti-inflammatory effects on macrophage	NK	[85]
	PVL	PC	in vitro: polymorphonuclear leukocytes in vivo: rabbit SAP, murine sepsis models	Neutralization of cytotoxic effects (IVIG); protective immunity	Important role in pathogenesis of necrotizing pneumonia	Low prevalence	[86,87,88,89]
	SEVs	PC	in vivo: murine models (SAP, systemic infection, skin infection, sepsis)	Induction of protective immunity	Nano-size; safety profile; multivalent nature; longer persistence in host; induction of innate/adaptive immune response; intrinsic adjuvanticity	Insufficient humoral response (reason for failure of passive immunization)	[90,91,92,93]
	LTA	PC	in vitro: cell cultures; in vivo: healthy humans	Neutrophil recruitment; proinflammatory; pro-apoptotic effects on macrophages; affects hemostasis	Crucial *S. aureus* component	Weak immunogenicity	[94,95,96,97]
No	SpA	PC	in vivo: murine models (skin abscess, sepsis)	Induction of protective immunity against *S. aureus*-induced infection	Induction of antigen-specific IgG response; protection from abscess formation and neonatal sepsis in mice; prevention of *S. aureus* colonization	Unexplored as single target	[98,99,100,101,102]
		C	Vaccination study in human (*S. aureus* bacteremia)	Good safety profile and minimal side effects in patients			
	FnBPA	PC	in vitro: cell culture in vivo: vaccination studies in mice (FnBPA/SpA bivalent fusion vaccine; murine pneumonia and bacteremia model)	Induction of protective immunity against *S. aureus*-induced infections; induction of *S. aureus* killing by neutrophils	Bivalent vaccine more promising than SpA alone	Unexplored as single target	[103]
	Fur	PC	in vivo: murine SAP model	*S. aureus* lacking Fur is less virulent and protected against killing by neutrophils	Regulates several immunomodulatory proteins	Not yet targeted	[104]
No	LukAB	PC	in vitro: cell cultures in vivo: murine immunization (sepsis)	Neutralization of cytotoxicity; prevention of cell lysis	Main contributor in human phagocyte killing	Not well described	[105,106,107]
	LukED	PC	in vitro: cell cultures	Induction of partial resistance to killing; functional inhibition of LukED	Highly conserved in epidemic MRSA lineages	NK	[108]

SAP: *S. aureus* pneumonia; Ab: Antibody; IVIG: Intravenous immunoglobulin; Hla: Alpha-toxin; Hlb: Beta-toxin; Hlg: Gamma-toxin; PSMs: Phenol soluble modulins; FnBPA: Fibronectin binding protein A; Spa: Staphylococcal protein A; SasX: *S. aureus* surface protein X; Srt: Staphylococcal sortases; PVL: Panton-Valentine leukocidin; SEVs: *S. aureus* extracellular vesicles; LTA: Lipoteichoic acid; LukAB: Leukocidin AB; LukED: Leukocidin ED; Fur: Ferric uptake regulator; NK: Not known. ^a^ PC: Preclinical; C: Clinical. ^b^ SEVs and OMVs: (Dis)advantages of therapeutic use.

**Table 3:** Clinical trials on *S. aureus* pneumonia.

Antigen(s)	Year	Type of Study ^a^	Study Title	No. of Subjects	Aim	Clinical Trials Identifier	Countries ^b^	Status of Trial	Outcome
SpA	2015	I, R	A I-II study of the safety and efficacy of a true human antibody, 514G3, in subjects sospitalized with bacteremia due to *S. aureus*	52	Evaluating the safety of 514G3 in patients with *S. aureus* bacteremia	NCT02357966	US	completed	Results ^c^
PVL	2016	O, NR	Panton-Valentine leucocidin: independent severity factor of *S. aureus* pneumonias	234	Assessing patient survival according to the PVL character of isolated *S. aureus* strains	NCT02798497	FR	completed	No published results
PVL	2017	O, Re	Epidemiology of post-influenza bacterial pneumonia due to a Panton-Valentine leukocidin positive *S. aureus* (FLUVALENTINE)	35	Evaluating the mortality of ICU patients with post-influenza bacterial pneumonia due to a PVL+ *S. aureus*	NCT03367624	FR	unknown	No published results
Hla	2019	I, R	A phase II randomized, double-blind, placebo-controlled, single-dose, dose-ranging study of the efficacy and safety of MEDI4893, a human monoclonal antibody against *S. aureus* alpha Toxin in mechanically ventilated adult subjects (SAATELLITE)	213	Studying the efficacy and safety of MEDI4893 (suvratoxumab)	NCT02296320	BE, CH, CZ, ES, FR, DE, GR, HU, PT, US	completed	Preliminary results ^d^ Results ^e^
Hla	2019	I, R	A randomized double-blind placebo-controlled multicenter phase III Study of efficacy and safety of AR-301 as adjunct therapy to antibiotics in the treatment of ventilator-associated pneumonia (VAP) caused by *S. aureus*	240	Testing of AR-301 as adjunctive to antibiotics in *S. aureus* VAP treatment	NCT03816956	BE, BR, BY, EE, FR, GE, IL, IN, LV, MX, RS, RU, TR, UA, US, ZA	recruiting	Trial currently ongoing
Hla, LukSF-PV, LukED, Hlg, LukGH	2019	I, R	A phase II, randomized, double-Blind, placebo-controlled study to determine the safety and efficacy of a single dose of ASN100 for the prevention of *S. aureus* pneumonia in heavily colonized, mechanically ventilated subjects	155	Assessing prevention of SAP in mechanically ventilated, heavily *S. aureus*-colonized subjects	NCT02940626	AT, CZ, ES, FR, GE, HU, IL, IN, PL, PT, RO, RS, RU, UA, US, ZA	completed	No published results
Hla	2020	I, R	A randomized, double-blind, placebo-controlled, single ascending dose study to assess the safety, pharmacokinetics, efficacy and pharmacodynamics of KBSA301 in severe pneumonia (*S. aureus*)	48	Evaluating the safety, pharmacokinetics and efficacy of KBSA301 in severe SAP	NCT01589185	BE, ES, FR, US	completed	Preliminary results ^f^

^a^ I: interventional, O: observational, R: randomized, NR: non-randomized, Re: retrospective. ^b^ BE: Belgium, BR: Brazil, BY: Belarus, CH: Switzerland, CZ: Czechia, DE: Germany, EE: Estonia, ES: Spain, FR: France, GE: Georgia, GR: Greece, HU: Hungary, IL: Israel, IN: India, PL: Poland, PT: Portugal, RO: Romania, RS: Serbia, RU: Russian Federation, UA: Ukraine, US: United States, ZA: South Africa. ^c^ doi: 10.1093/ofid/ofw172.1057. ^d^ doi: 10.1128/AAC.01020-16. ^e^ https://www.clinicaltrialsregister.eu/ctr-search/trial/2014-001097-34/results. ^f^ doi: 10.1007/s00134-018-5229-2.

**Table 4:** Current issues hampering *S. aureus* vaccine research and alternative therapeutic strategies.

**(** **A) Issues Hampering *S. aureus* Vaccine Research**	**Potential Solution**	**References**
Redundancy of *S. aureus* virulence factors	Target regulatory factors	[83,201,208]
Genetic variations among *S. aureus* isolates/lineages		
Complex regulatory mechanisms		
Lower presence and expression of virulence factors in HA-MRSA		[34,35]
Antibody-based therapy less effective in highly colonized SAP patients	Prophylactic antibody use to be explored	[209]
High tropism of *S. aureus* virulence factors Inferior transferability of conventional mouse models into clinical research	Humanized mice	[194,210,211,212,213,214,215] 10.3390/ijms21197061
	Mouse-adapted *S. aureus* strains	
	Recombinant toxins	
**(B) Alternative Therapeutic Strategies**	**Explored in SAP**	**References**
Bacteriophages	Yes	[216,217,218,219,220,221,222,223,224]
Outer membrane vesicles	No	[225]
Nanoparticles (nasal vaccination)	No	10.1016/j.addr.2008.09.005
Nanoparticles (treatment of pulmonary diseases)	Yes	10.1002/wnan.1401
		10.1038/s41551-017-0187-5
Antimicrobial peptides (antibiotic alternative)	No	10.1093/jac/dkw381

HA-MRSA: Hospital-associated methicillin-resistant *S. aureus*; SAP: *S. aureus pneumonia.*
